# Obstetric Aphorisms

**Published:** 1881-11-20

**Authors:** H. Webster Jones

**Affiliations:** Chicago


### OBSTETRIC APHORISMS.

BY H. WEBSTER JONES, M. D., CHICAGO.

1. An intelligent confidence once thoroughly established between patient and physician does much to banish the terrors of the lying-in-room.

2. It is possible to foresee and prevent the occurrence of the almost fatal form of eclampsia gravidarum.

3. Cleanliness is especially next to godliness in the case of the accoucheur. Its absence renders one liable to professional homicide.

4. The modern midwifery must not be meddlesome, but must be mediatorial in the sense of palliating suffering, expediting nature's processes by well proven means, and removing scientifically all the inexplicable, accidental or morbid states and conditions. Idleness is no longer an approved qualification for a degree of obstetrics.

5. The hand is the best uterine dilator.

6. The forceps should never be employed until the os uteri is dilated or dilatable, and then not unless the membranes have been ruptured and labor delayed unnaturally for at least an hour. Every practitioner should become skillful in their use, and they should never be left at home for fear of temptation.

7. Unnecessary and avoidable delays in labor are fruitful sources of gynecological practice. They promote inflammation and sepsis.

8. The patient's hopeful confidence and the physician's industrious attention, actually contribute to the physiological elements of labor. Anæsthetics here are, to say the least, superfluous.

9. Bi-manual aid in effecting the deliverance of the placenta, is not only proper but advisable. Skillfully rendered, the cry of "uterine inversion" becomes no longer a bug-bear.

10. The continuous and intelligent counter-pressure over the fundus uteri during the child's exit, the delivery of the placenta and the period of frequent oscillation, be that a shorter or a longer time, is a safeguard never to be neglected.

11. Pursuant to the same end, the application of the bandage and its continuance, as long as the uterine globe can be felt and embraced by it above the pubis, contributes not only to comfort, but to speedy involution. After the seventh day, close pressure must be interdicted.

12. Puffiness of one ankle, with tenderness of the corresponding groin, and an abnormally quickened pulse, with or without copious sweating, noticed within the first ten days after labor, betoken the presence of phlebitis, and the possibility of embolism or thrombus, and resultant sudden death.

13. The duties of an obstetrician are not concluded until a careful examination, from six to eight weeks after parturition, proves the integrity of all the organs concerned.—*Mich. Med. News.*

